# Supplementary material for: Molecular insights into the capsular polysaccharide transporter Wza-Wzc complex
Source: Nat Commun. 2026 Feb 5;17:1436. doi: 10.1038/s41467-026-69136-2 (PMC12881504; doi:10.1038/s41467-026-69136-2)
Supplement: Supplementary file 1 — Supp. Information [file 41467_2026_69136_MOESM1_ESM.pdf]

SUPPLEMENTARY INFORMATION

Molecular insights into the capsular polysaccharide transporter Wza-Wzc complex

Biao Yuan[1,@], Christian Sieben[2], Prateek Raj[1], Tina Rietschel[3], Rory Hennell James[4,5,6], Anja Gatzemeier[1], Lothar Jänsch[3], Thomas C. Marlovits[4,5,6], Dirk W. Heinz[1,@]

SUPPLEMENTARY FIGURES

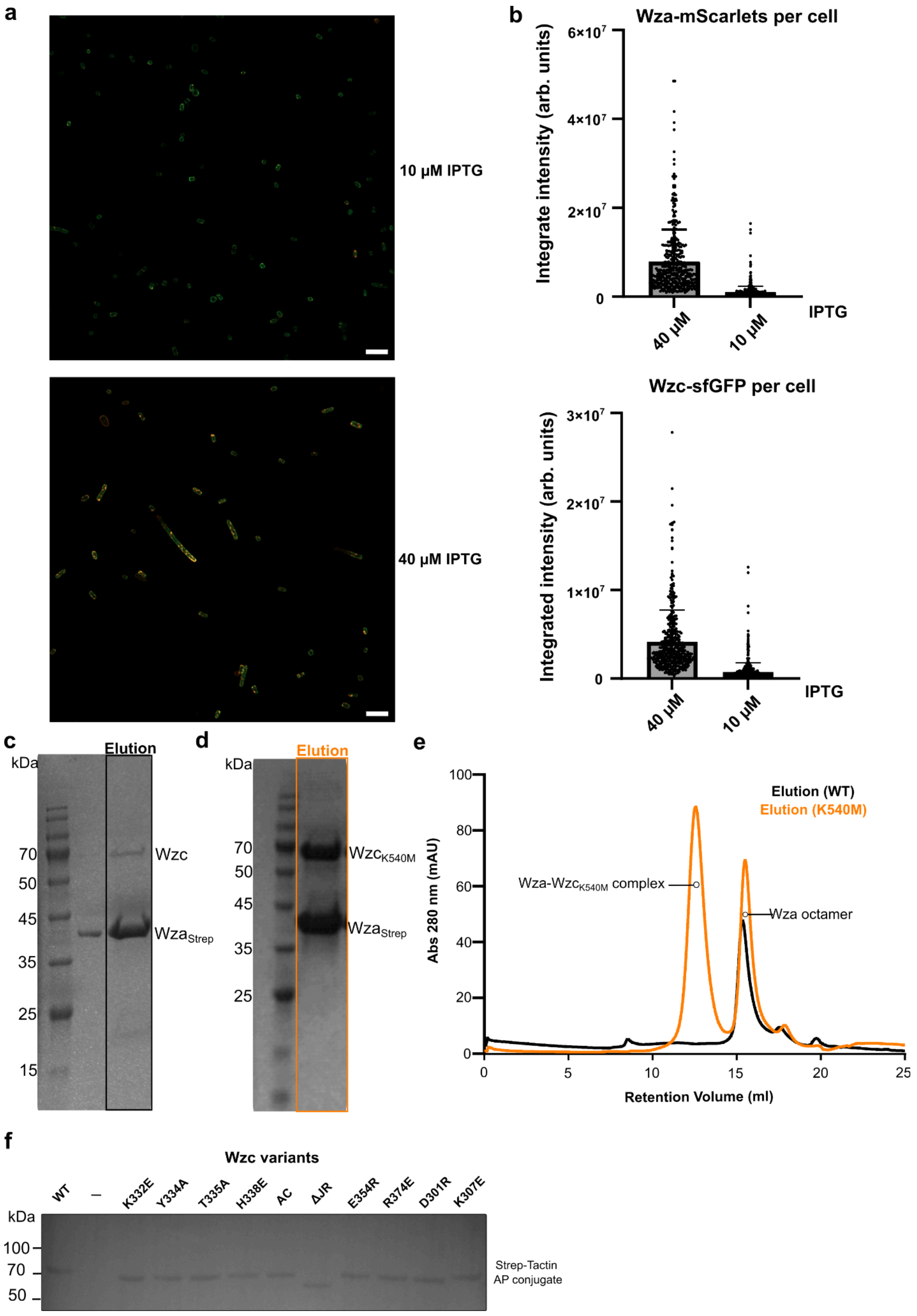

Supplementary Fig. 1. Single-cell quantification of protein expression and purification of the Wza-Wzc complex and western-blot analysis of the expression level of Wzc variants. Single-cell quantification of protein expression after IPTG induction **(a,b)**. Following induction with IPTG at the indicated concentrations for 16 hours at 19°C, we imaged the cells using confocal fluorescence microscopy (**a**); Scale bar: 5 μm. To quantify the fluorescence signal per cell, the signal of Wzc-sfGFP was first used to create a binary mask in Fiji/ImageJ, which was then used to segment individual cells from each micrograph. The integrated intensity per individual cell was then quantified for each cell in both channels using Fiji/ImageJ. The results are shown in **b**. arb. units: arbitrary units. **(c)** SDS-PAGE analysis shows the elution profile of *E. coli* BL21 Star (DE3) cells overexpressing the full *wzabc* operon with C-terminal Strep-tag on Wza from Strep-tactin XT resin. A little amount of wild-type Wzc was co-purified with Wza_{Strep}. **(d)** The octameric variant Wzc_{K540M} was co-purified with Wza_{Strep}. SDS-PAGE analysis of elution from *E. coli* BL21 Star (DE3) expressing the full *wzabc* operon with C-terminally Strep-tagged Wza and K540M variant of Wzc. **(e)** Size-exclusion chromatography (SEC) profiles of the elution samples from panels **c** and **d**. The Wza-Wzc_{K540M} complex purified by SEC was used for subsequent cryo-EM analysis. mAU: milli–absorbance units. **(f)** Western blot analysis of Wzc variant expression levels corresponding to the samples used in the CPS production assay. Cell cultures were normalized to equal cell densities based on OD_{600} measurements during SDS-PAGE sample preparation. Strep-Tactin AP conjugate was used to detect the Wzc-strep variants.

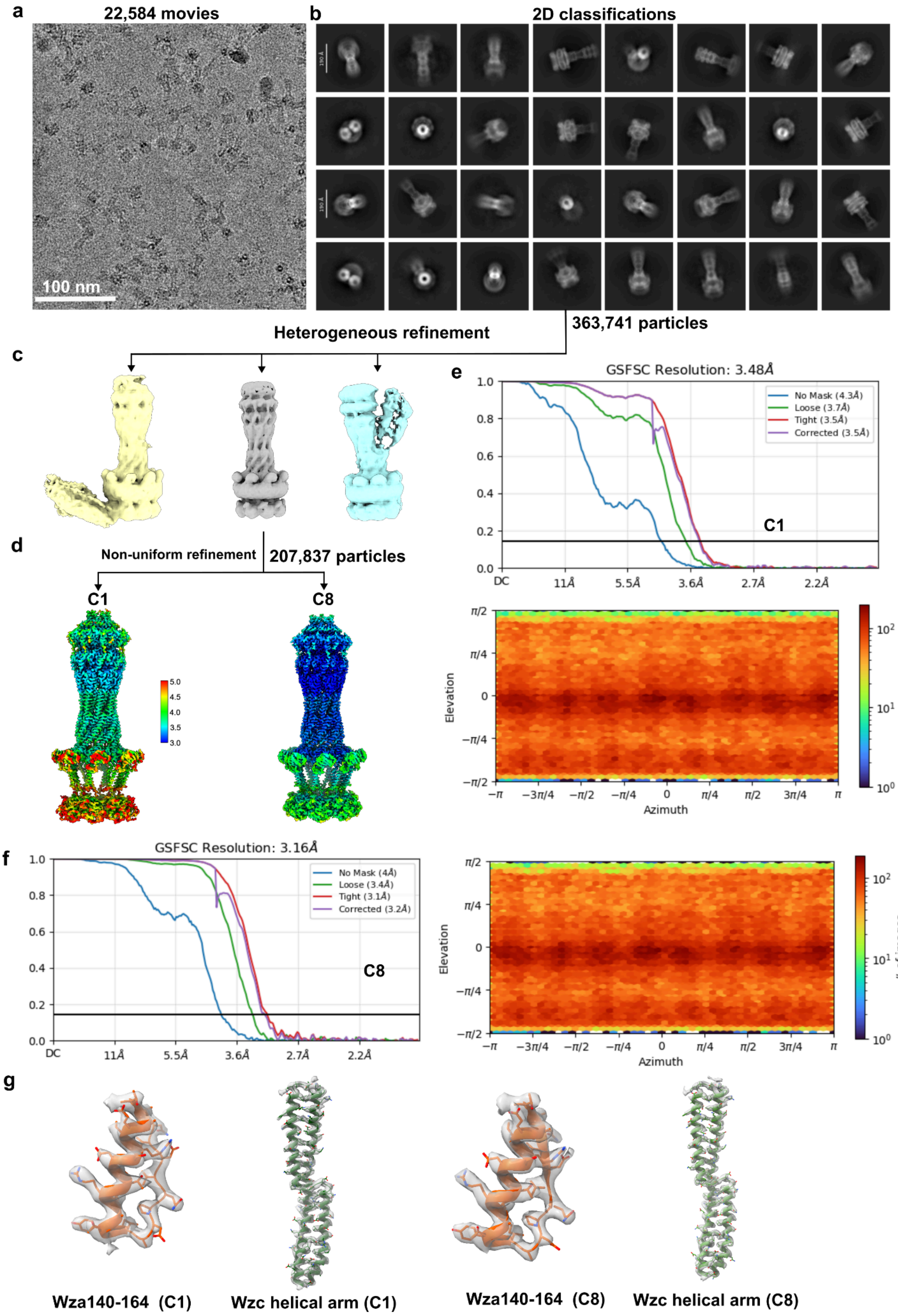
a
22,584 movies
100 nm
b
2D classifications
363,741 particles
Heterogeneous refinement
c
d
Non-uniform refinement
207,837 particles
C1
C8
e
GSFSC Resolution: 3.48Å
No Mask (4.3Å)
Loose (3.7Å)
Tight (3.5Å)
Corrected (3.5Å)
C1
Elevation
Azimuth
# of images
f
GSFSC Resolution: 3.16Å
No Mask (4Å)
Loose (3.4Å)
Tight (3.1Å)
Corrected (3.2Å)
C8
g
Wza140-164 (C1)
Wzc helical arm (C1)
Wza140-164 (C8)
Wzc helical arm (C8)

Supplementary Fig. 2. Cryo-EM data processing workflow for the Wza-Wzc$_{K540M}$ complex. **(a)** Representative cryo-EM micrograph of the Wza-Wzc$_{K540M}$ complex. Scale bar: 100 nm. **(b)** Selected 2D class averages used for subsequent analysis. Scale bar: 100 Å. **(c)** 3D classification step to exclude poor-quality or junk particles. **(d)** Final cryo-EM density maps generated using non-uniform refinement under C1 and C8 symmetries, colored according to local resolution. **(e)** Gold standard Fourier Shell Correlation (FSC) curve and orientation distribution of particles for the refined C1 cryo-EM map. **(f)** FSC curve and orientation distribution of particles for the refined C8 cryo-EM map. **(g)** Representative cryo-EM density maps superposed with the atomic models.

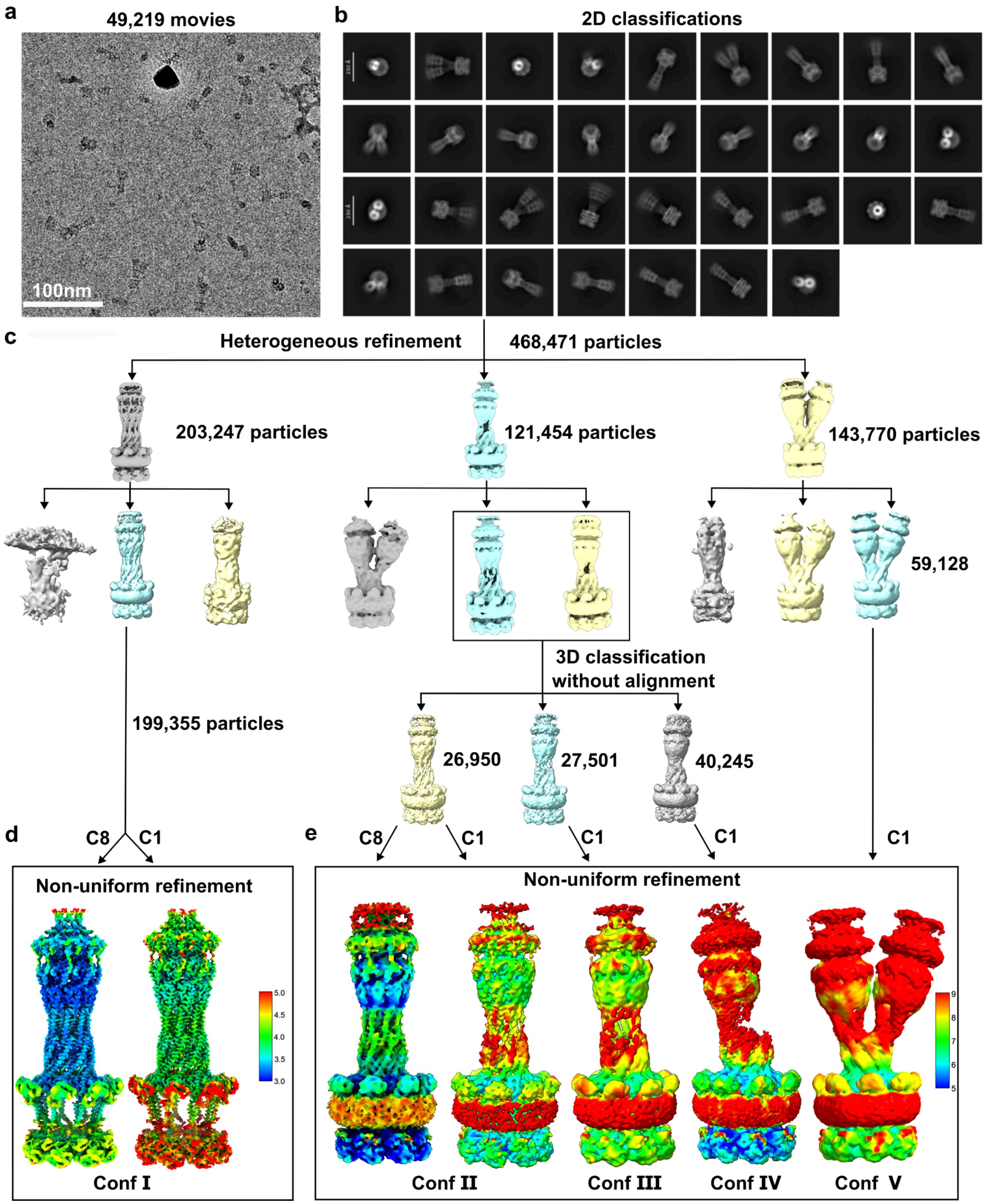

Supplementary Fig. 3. Data processing of the EDTA-treated Wza-Wzc$_{K540M}$ complex. (a) Representative cryo-EM micrograph of the Wza-Wzc$_{K540M}$ complex following treatment with 10 mM EDTA. **(b)** Selected 2D class averages used for subsequent 3D classification. **(c)** Workflow involved two rounds of 3D classification, non-uniform refinement, and alignment-free 3D

classification to resolve multiple conformational states of the complex. **(d-e)** Five distinct conformational states (Confs Ⅰ – Ⅴ) were identified, with corresponding density maps colored according to local resolution.

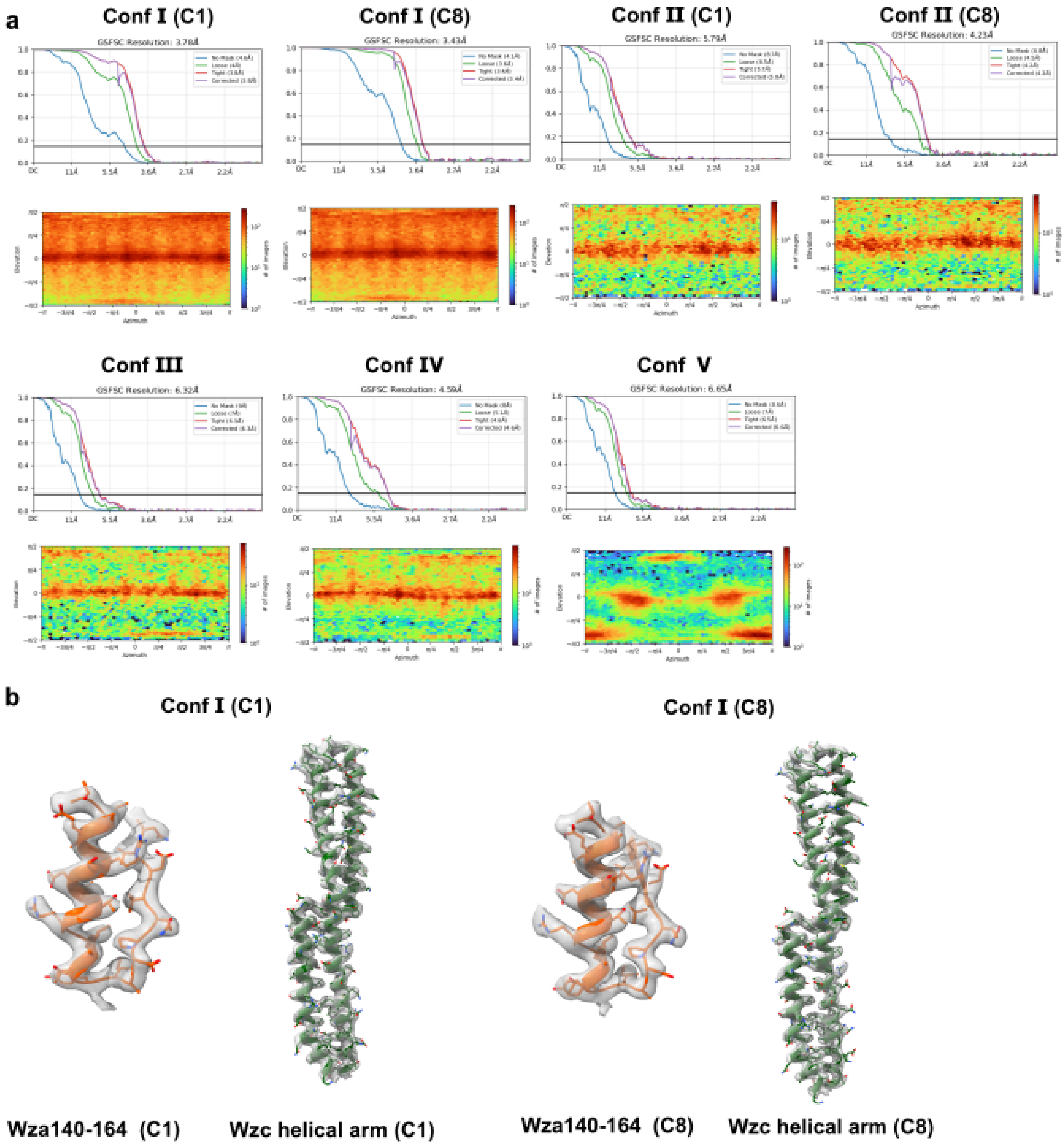

Supplementary Fig. 4. cryo-EM data of the EDTA-treated Wza-Wzc$_{K540M}$ complex. (a) FSC curves and orientation distributions of particles for the cryo-EM maps of different conformational

states of the Wza-Wzc complex. **(b)** Representative cryo-EM density maps superposed with the atomic models.

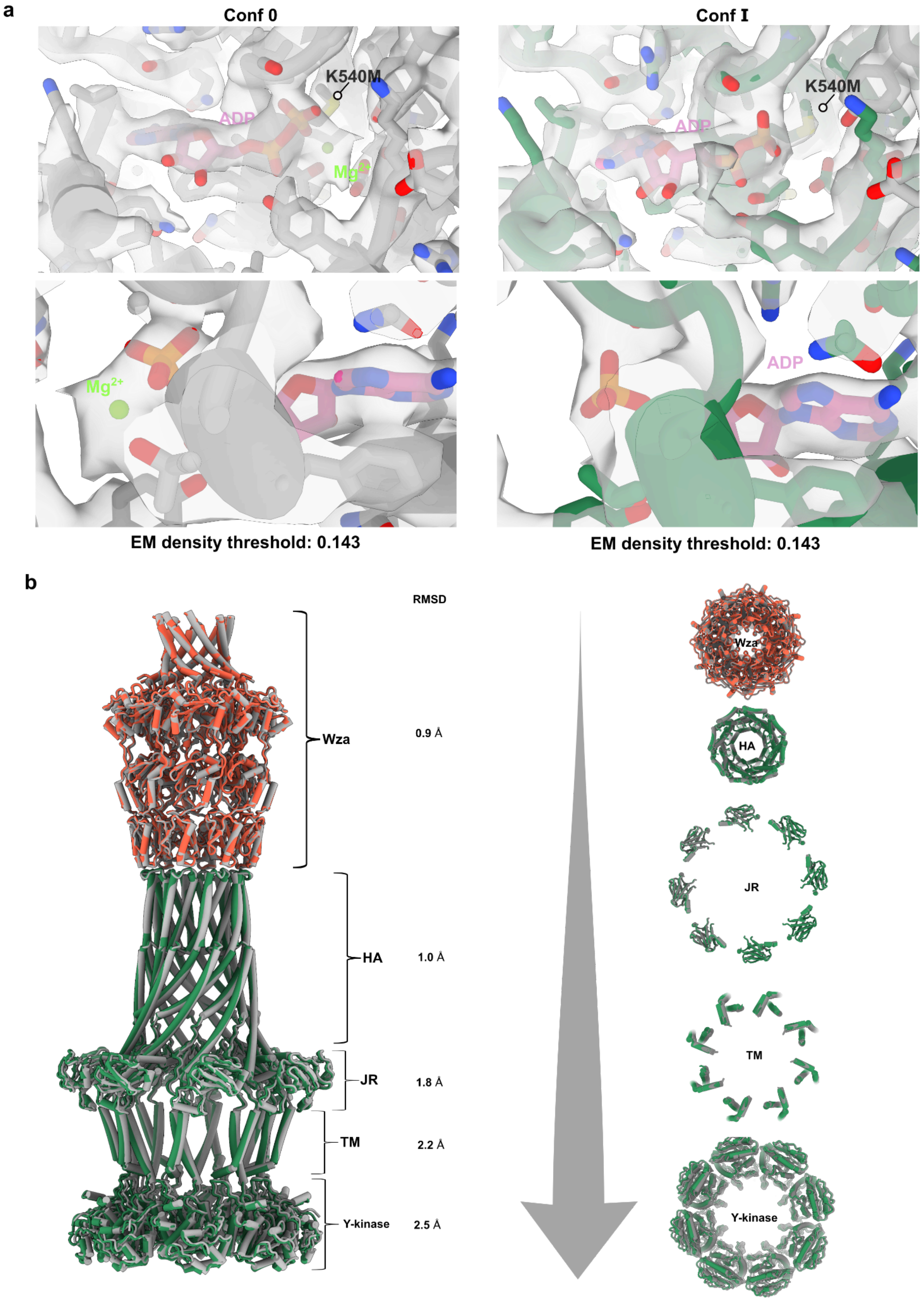
a
Conf 0
Conf I
K540M
ADP
Mg2+
K540M
ADP
Mg2+
ADP
EM density threshold: 0.143
EM density threshold: 0.143
b
RMSD
Wza
0.9 Å
HA
1.0 Å
JR
1.8 Å
TM
2.2 Å
Y-kinase
2.5 Å
Wza
HA
JR
TM
Y-kinase

Supplementary Fig. 5 Structural comparison between Conf Ⅰ and Conf 0 of the Wza-Wzc complex. (a) Cryo-EM maps of the ADP-Mg^{2+} binding pockets in the Wza-Wzc_{K540M} complex. The Conf 0 structure (shown in **Fig. 2c)** is colored grey while the Conf Ⅰ structure (shown in **Fig. 3b**) is overlaid for comparison. Two distinct views are shown for each EM map at an identical volume threshold. **(b)** Structural superposition of Conf Ⅰ and Conf 0 of the Wza-Wzc_{K540M} complex aligned on the Wza translocon, including local RMSD values and cross-sectional views highlighting differences in Wza and the individual subdomains of Wzc.

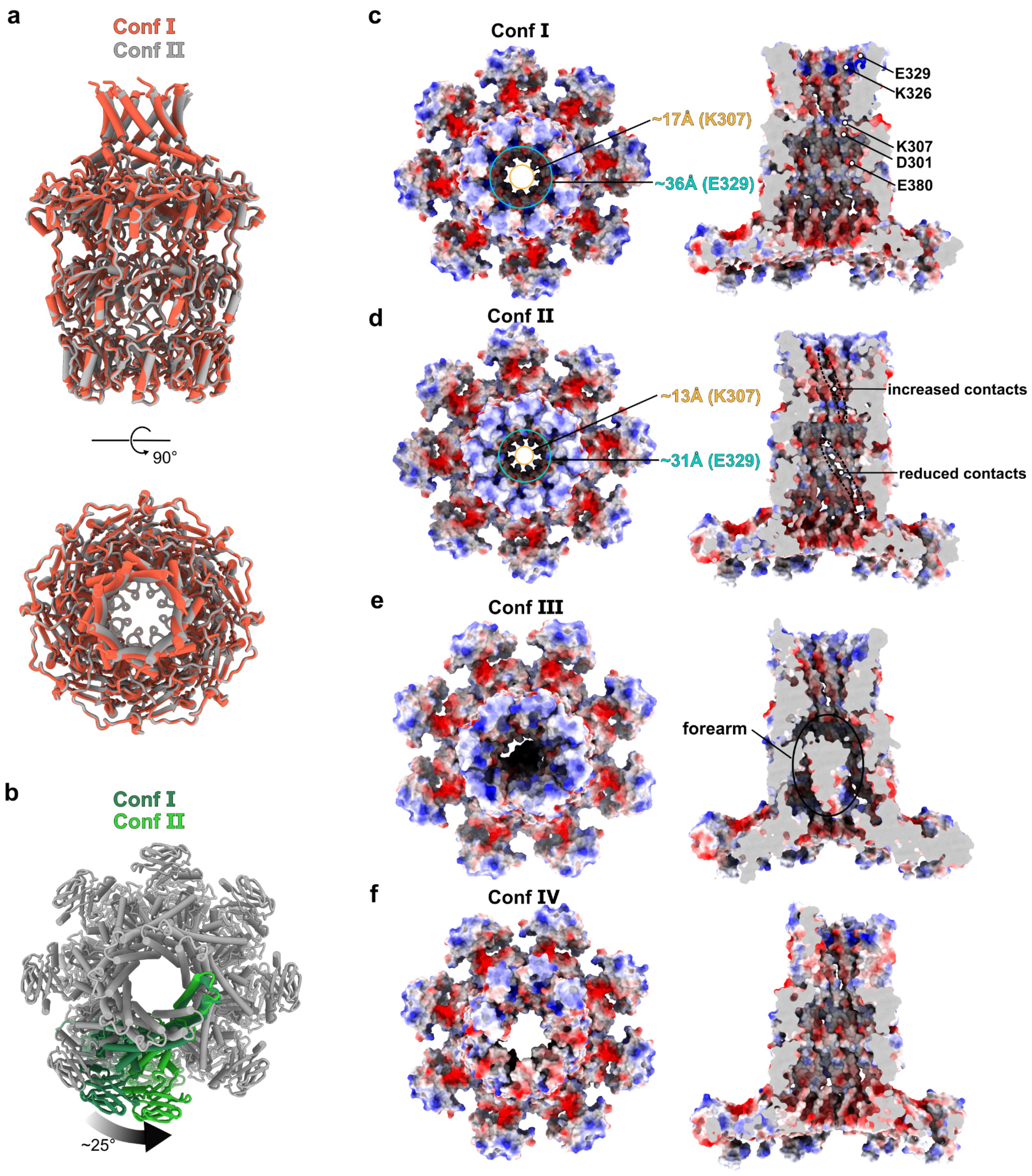

Supplementary Fig. 6. Structural and electrostatic changes of the Wzc periplasmic channel across distinct conformational states. (a) Structural superposition of the Wza part of Conf Ⅰ (in red) and Conf Ⅱ (in grey) states of the Wza-Wzc$_{K540M}$ complex. **(b)** Structural superposition of Conf Ⅰ and Conf Ⅱ states of the Wza-Wzc$_{K540M}$ complex, aligned on the Wza translocon to highlight the potential rotation mechanism of Wzc. **(c–f)** Electrostatic surface

representations and cross-sectional views of the Wzc periplasmic domain are shown for Confs Ⅰ – Ⅳ. **(c)** Conf Ⅰ exhibits an open channel with a lumen diameter of ~17 Å at K307 and ~36 Å at E329. Positively and negatively charged residues (K307, D301, K326, E329, E380) line the pore, forming a balanced electrostatic environment. **(d)** In Conf Ⅱ, the channel narrows (~13 Å at K307 and ~31 Å at E329) and displays rearranged charge contacts at the AC interface, reflecting a more compact channel region formed by forearms. While the channel region formed by upper arms is less compacted. **(e)** Conf Ⅲ reveals the sealed channel with its forearm domain. **(f)** Conf Ⅳ shows partial channel collapse with the remaining four forearms connecting with the Wza translocon, indicating a pre- or post-translocation state.

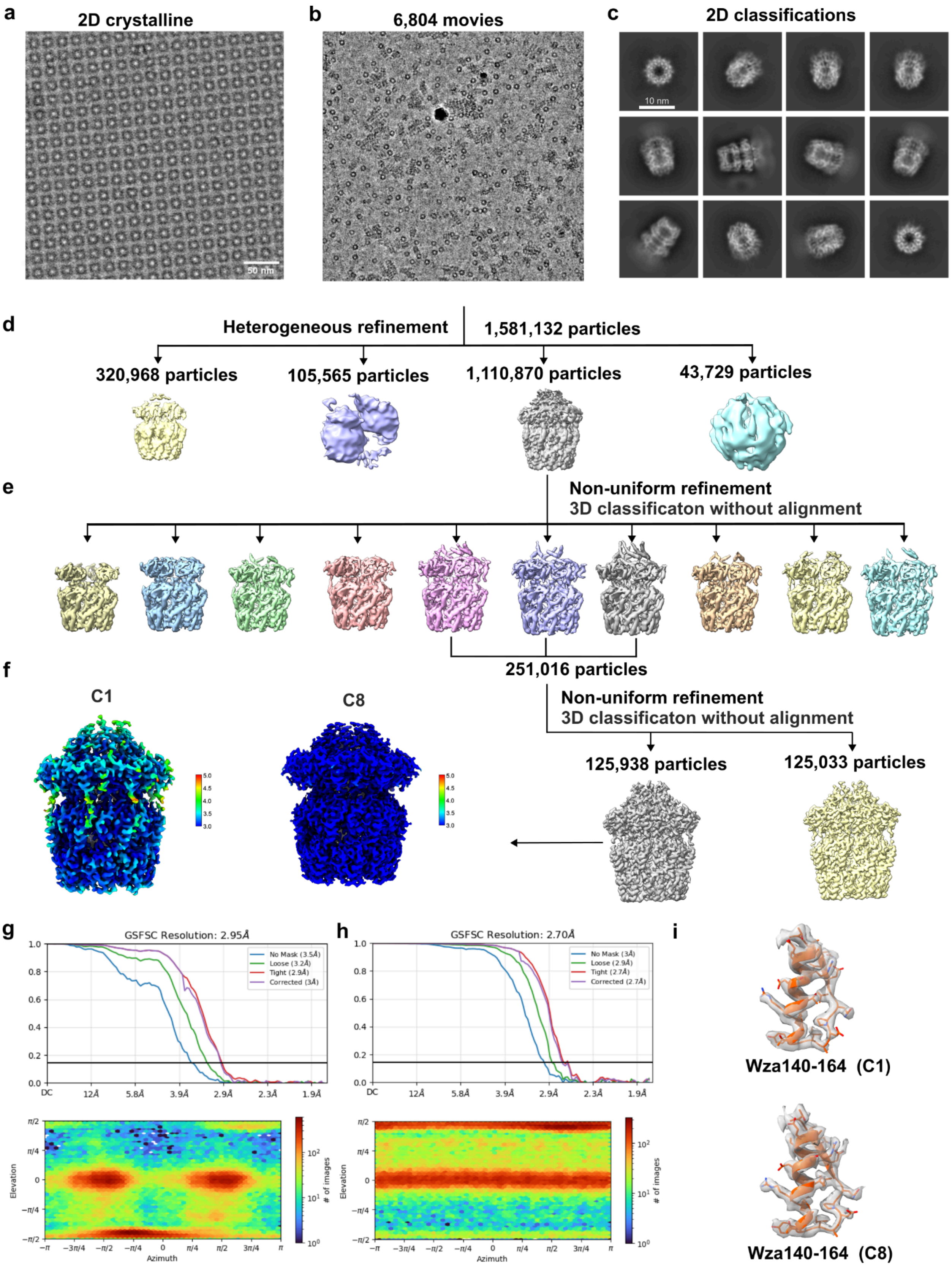
a
2D crystalline
50 nm
b
6,804 movies
c
2D classifications
10 nm
d
Heterogeneous refinement
1,581,132 particles
320,968 particles
105,565 particles
1,110,870 particles
43,729 particles
e
Non-uniform refinement
3D classificaton without alignment
251,016 particles
f
C1
C8
Non-uniform refinement
3D classificaton without alignment
125,938 particles
125,033 particles
g
GSFSC Resolution: 2.95Å
No Mask (3.5Å)
Loose (3.2Å)
Tight (2.9Å)
Corrected (3Å)
h
GSFSC Resolution: 2.70Å
No Mask (3Å)
Loose (2.9Å)
Tight (2.7Å)
Corrected (2.7Å)
Elevation
Azimuth
# of images
i
Wza140-164 (C1)
Wza140-164 (C8)

Supplementary Fig. 7. Data processing workflow for the Wza octamer. (a) Wza tends to form two-dimensional crystalline arrays in solution. **(b)** Representative cryo-EM micrograph of the Wza octamer. **(c)** Selected high-quality 2D class averages used for further analysis. **(d)** Initial 3D classification to eliminate junk and broken particles. **(e)** Two rounds of non-uniform refinement and alignment-free 3D classification to further remove broken particles. **(f)** Final cryo-EM maps of the isolated Wza octamer. **(g)** FSC curve and orientation distribution of particles obtained from non-uniform refinement with C1 symmetry. **(h)** FSC curve and orientation distribution of particles obtained from non-uniform refinement with C8 symmetry. **(i)** Representative cryo-EM density maps superposed with the atomic models.

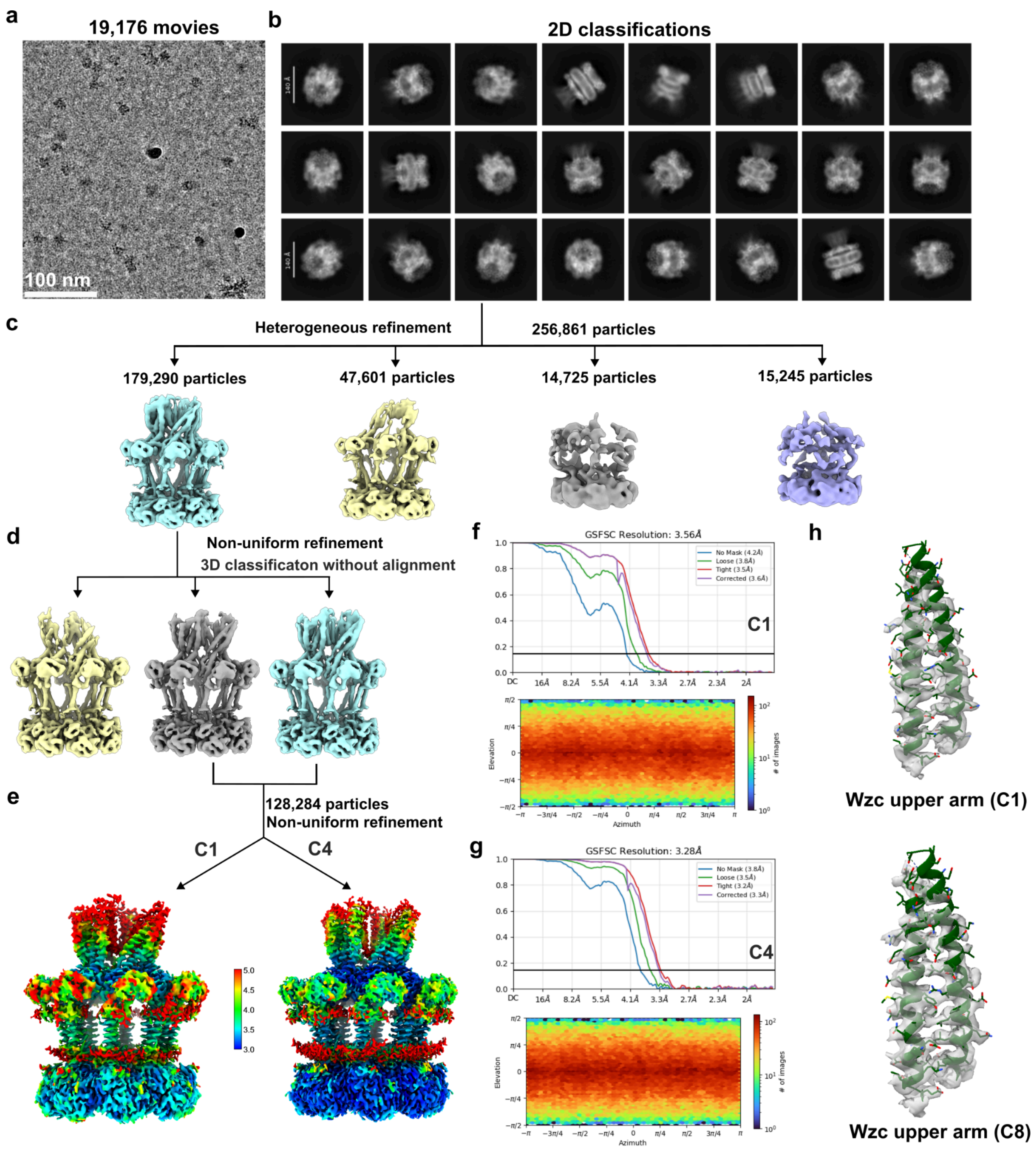

Supplementary Fig. 8. Data processing workflow for the Wzc_{K540M} octamer. (**a**) Representative cryo-EM micrograph of the Wzc_{K540M} octamer. (**b**) Selected high-quality 2D class averages from 2D classifications. (**c**) 3D classification step used to remove junk classes. (**d**)

One round of non-uniform refinement followed by alignment-free 3D classification to eliminate remaining low-quality classes. (**e**) Final cryo-EM density maps obtained from non-uniform refinements with C1 and C4 symmetries applied. (**f**) FSC curve and orientation distribution for the C1-refined map. (**g**) FSC curve and orientation distribution for the C4-refined map. **(h)** Representative cryo-EM density maps superposed with the atomic models.

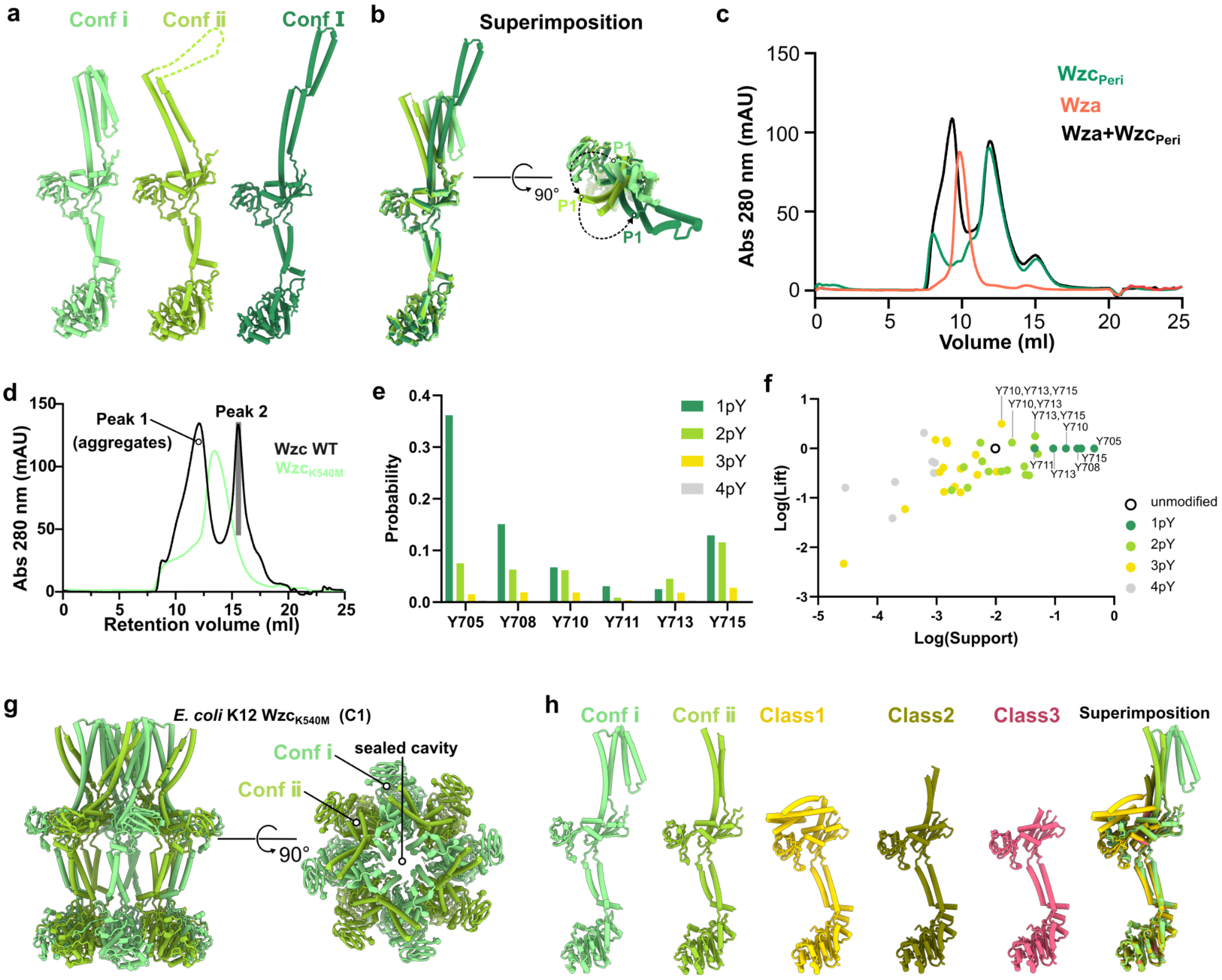

Supplementary Fig. 9. Structural comparison between *E. coli* K12 and K30 $\text{Wzc}_{\text{K540M}}$ structures. (a) Structural comparison of protomers of the Wzc octamer and the Wza-$\text{Wzc}_{\text{K540M}}$ complex. (**b**) Superimposition of these protomers indicating the trajectory of HA to recruit the Wza translocon. (**c**) SEC profiles of Wzc_{Peri}, Wza alone, and a combined sample of Wzc_{Peri} and Wza, with the mixture containing equal amounts of each protein as used separately. mAU:

milli–absorbance units **(d)** SEC profiles of purified wild-type Wzc and Wzc_{K540M}. Peak 1 sample of wild-type Wzc corresponds to soluble aggregates, while Peak 2 represents a lower-order oligomeric species. Separation was performed using a Superose 6 Increase 10/300 GL column following affinity purification with a Strep-Tactin-XT column. mAU: milli–absorbance units **(e)** Phosphorylation probability of each tyrosine position at Y-tails with 1pY, 2pY, 3pY, and 4pY. **(f)** Association analysis of different phosphorylated sites at Y-tails phosphorylation sites indicating the prevalence (Support) and co-occurence (Lift) of single or higher order phosphorylations. **(g)** Cryo-EM structure of *E. coli* K12 Wzc_{K540M} (C1). (**h**) Structural comparison of different states of the protomers of *E. coli* K12 and K30 Wzc_{K540M} structures[1]. Conf i , Wzc protomer at Conf i state from the Wzc_{K540M} octamer; Conf ii , Wzc protomer at Conf ii state from Wzc_{K540M} octamer; Conf I , Wzc protomer from the Wza-Wzc_{K540M} complex at Conf I state. Superposition of these Wzc protomers showing HA under large conformational changes.

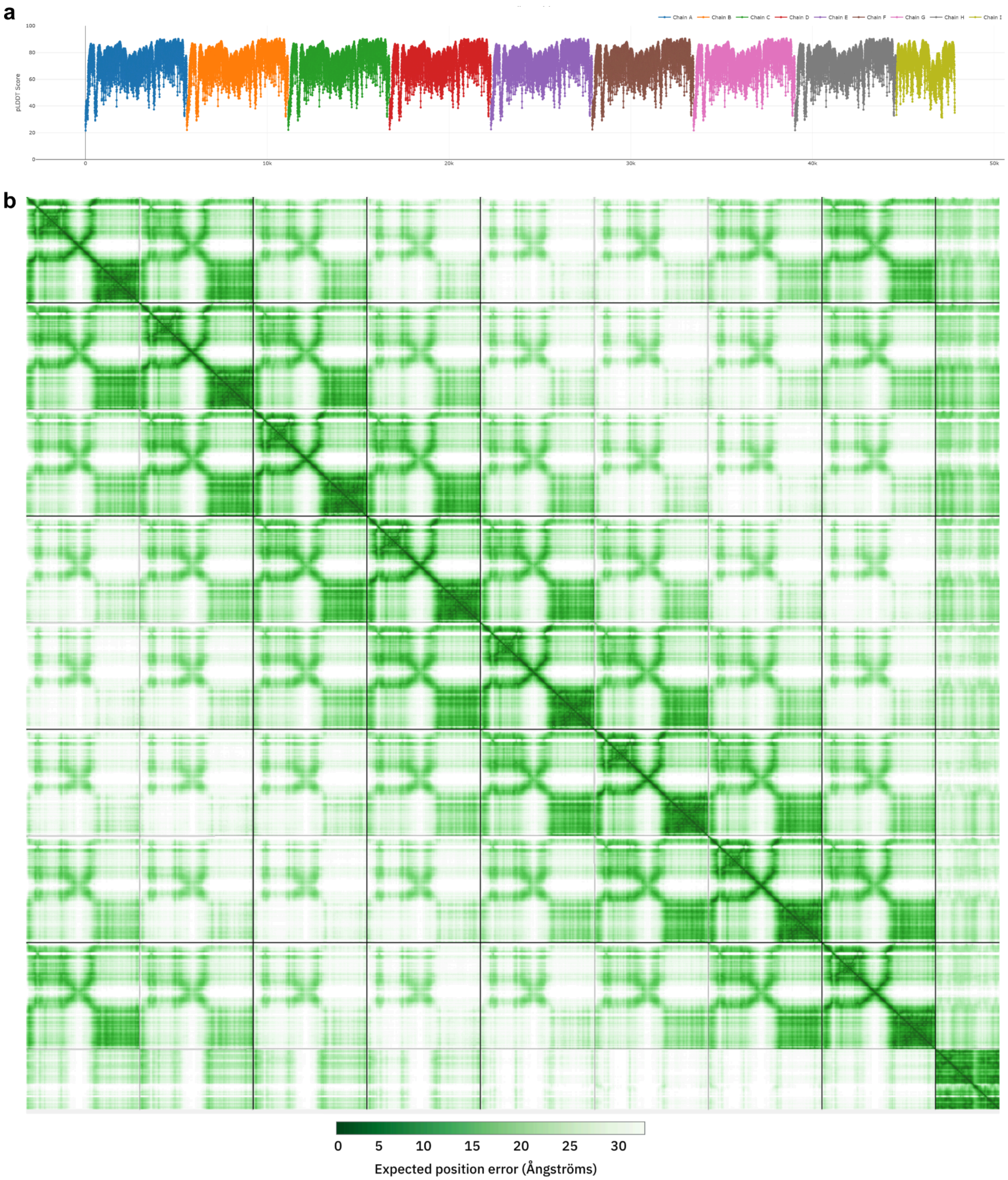

Supplementary Fig. 10. Predicted pLDDT and PAE metrics for the AlphaFold3-generated model of the Wzc(8)–Wzy(1) complex. (a) The pLDDT plot of Wzc-Wzy complex. Chain A-H, Wzc; Chain I, Wzy. **(b)** The PAE metrics of the Wzc-Wzy complex.

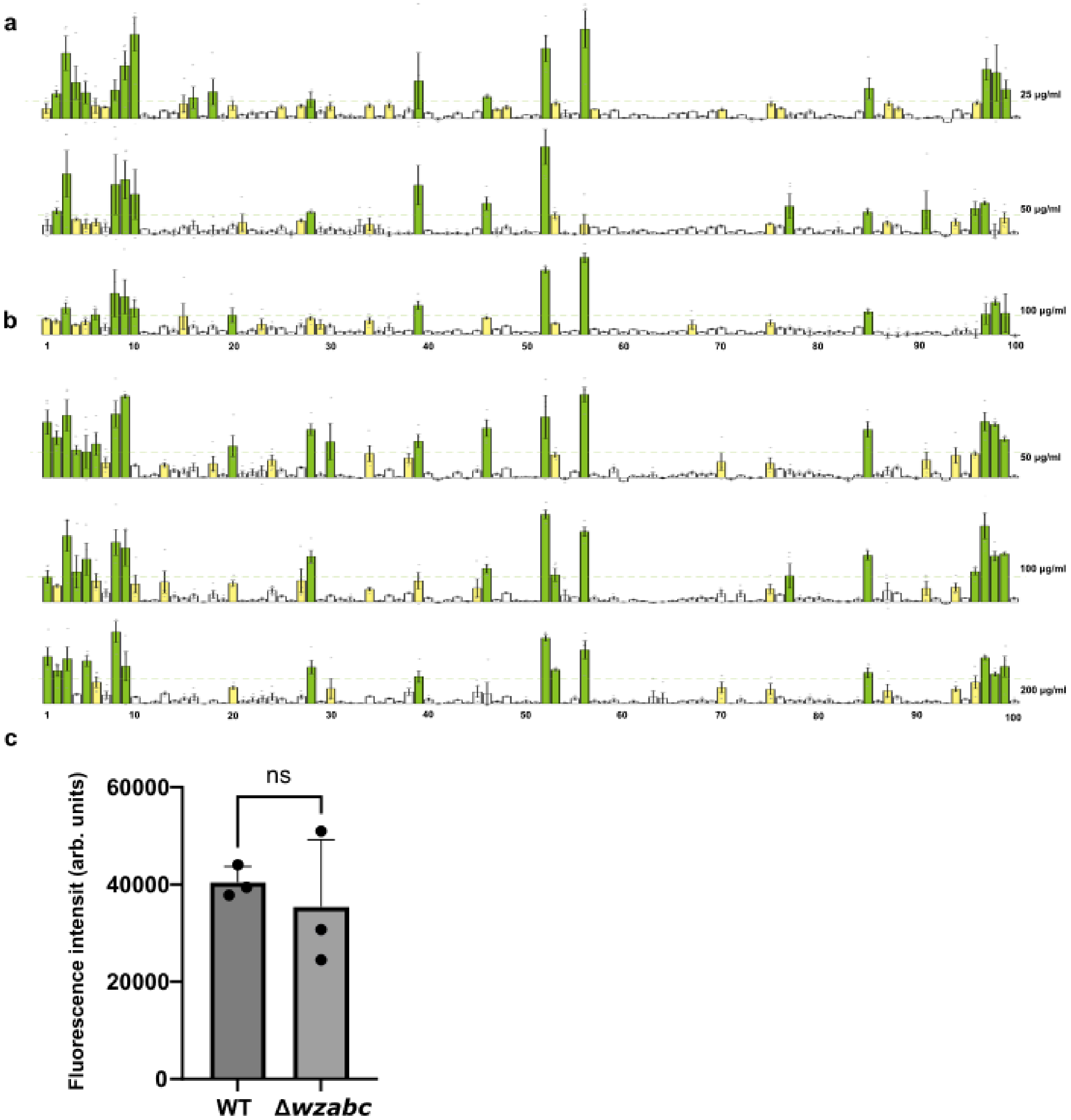

Supplementary Fig. 11. Glycan array and co-sedimentation assay. (a-b) Glycan binding profiles of Wzc_{JR} (a) and Wzc_{Peri} (b) at three different concentrations, as labels for each panel. Glycan-binding profile of biotinylated proteins detected using Cy3-conjugated streptavidin on the Glycan 100 array. The Y-axis represents relative fluorescence intensity, indicating the extent of protein binding to each glycan. The X-axis corresponds to glycan ID numbers as defined in the Glycan 100 array. The bars are colored according to the values of the relative fluorescence intensity (intensity>1000, green; 500<intensity<1000, yellow; intensity<500, white). Data are

presented as mean values +/- SD. Each spot represents one technical repeat. (**c**) Fluorescence intensity of *E. coli* after co-sedimentation with sfGFP-Wzc_{JR}. WT: *E. coli* JM109 (DE3) cells. Δ*wzabc*: *E. coli* JM109 (DE3)Δ*wzabc* cells, which lack colanic acid production. Measurements were performed in triplicate (n=3). P = 0.5952 arb. units: arbitrary units.

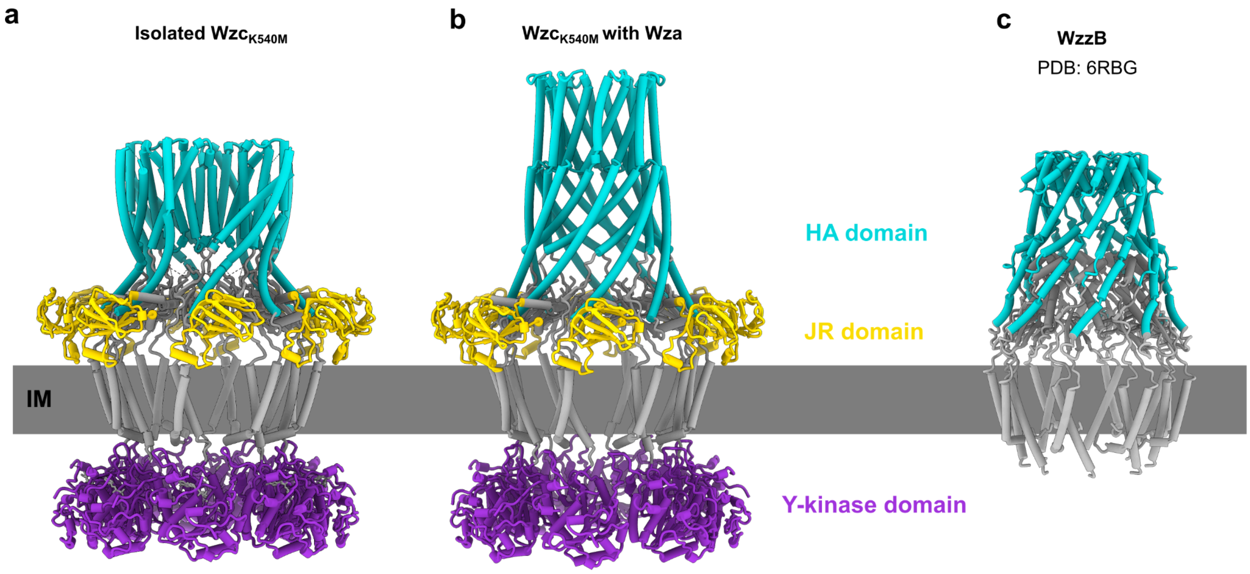

Supplementary Fig. 12. Structural comparison between Wzc and WzzB. (a) Model of the isolated Wzc_{K540M} octamer. **(b)** Model of the Wzc_{K540M} when bound to the Wza translocon. **(c)** Model of the full-length WzzB octamer[2].

SUPPLEMENTARY TABLES

Supplementary Table 1. Mass spectrometry analysis of the phosphorylation pattern on the Y-tail of Wzc.

Peptide sequence with Phospho (STY) Probabilities	PEP	Score	Intensity	Phosphorylation sites
ASAY(1)QDY(1)GY(1)YEYEYK	0	319,37	5965600	Y705, Y708, 710Y
ASAY(1)QDY(0.027)GY(0.472)Y(0.472)EY(0.009)EY(0.019)KSDAK	0	251,75	1,32E+08	Y705
ASAYQDYGY(0.034)Y(0.966)EY(1)EY(0.988)KS(0.013)DAK	0	301,03	2023000	YY08, Y710, Y711
ASAYQDYGY(0.026)Y(0.974)EY(0.995)EY(0.004)KSDAK	0	325,11	5682000	Y708, Y710
ASAY(0.006)QDY(0.994)GY(0.067)Y(0.933)EYEYK	0	275,17	9026500	Y708, Y711
ASAYQDY(0.031)GY(0.857)Y(0.109)EY(0.079)EY(0.902)KS(0.022)DAK	0	254,83	84666000	Y708, Y713
ASAYQDY(0.005)GY(0.994)Y(0.001)EYEYK	0	283	52737000	Y708
RAS(0.648)AY(0.352)QDYGY(0.137)Y(0.863)EY(0.027)EY(0.965)KS(0.007)DAK	0	266,78	1142400	Y710, Y713
RAS(0.622)AY(0.353)QDY(0.025)GY(0.858)Y(0.141)EY(0.002)EY(0.996)KS(0.003)DAK	0	240,82	4314600	Y710, Y715
ASAY(0.001)QDY(0.826)GY(0.155)Y(0.062)EY(0.962)EY(0.967)KS(0.026)DAK	0	259,82	29923000	Y713, Y715
RAS(0.427)AY(0.57)QDY(0.14)GY(0.839)Y(0.028)EY(0.028)EY(0.967)KS(0.001)DAK	0	254,16	3360500	Y715
ASAY(1)QDY(1)GYYEY(0.033)EY(0.967)K	0	258,53	3693200	Y705, Y708, Y711
AS(0.038)AY(0.962)QDY(1)GY(0.295)Y(0.705)EY(0.049)EY(0.946)KS(0.005)DAK	0	270,75	1817000	Y705, Y708, Y715
ASAY(1)QDYGY(1)YEYEYK	0	310,01	23300000	Y705, Y708
ASAY(1)QDYGYYEY(0.028)EY(0.972)K	0	364,34	19400000	Y705, Y710
RASAYQDYGY(0.937)Y(0.063)EY(0.005)EY(0.995)KS(0.001)DAK	0	252,44	5106400	Y705, Y711
ASAYQDYGY(0.757)Y(0.298)EY(0.917)EY(0.027)KS(0.001)DAK	3,4E-283	235,85	5315800	Y710
RAS(0.219)AY(0.781)QDY(1)GY(0.984)Y(0.016)EYEY(0.99)KS(0.01)DAK	7,5E-280	245,66	1511900	Y708, Y710, Y713
RAS(0.22)AY(0.782)QDY(0.999)GY(0.984)Y(0.016)EY(0.001)EY(0.989)KS(0.01)DAK	7,5E-280	245,66	1784500	Y708, Y710, Y715
RASAY(0.001)QDY(0.982)GY(0.015)Y(0.001)EY(0.001)EY(0.988)KS(0.01)DAK	7,3E-255	238,79	4371000	Y708, Y715
ASAY(1)QDY(0.999)GY(0.01)Y(0.135)EY(0.857)EY(0.956)KS(0.044)DAK	2,6E-254	230,27	5329800	Y705, Y708, Y713, Y715

ASAY(0.01)QDY(0.989)GY(0.924)Y(0.08)EY(0.996)EY(0.973)KS(0.027)DAK	5,2E-228	229,86	1246900	Y708, Y710, Y713, Y715
ASAYQDY(0.006)GY(0.609)Y(0.391)EY(0.993)EY(0.969)KS(0.032)DAK	1,7E-227	214	41992000	Y711, Y713
ASAY(0.001)QDY(0.98)GY(0.228)Y(0.804)EY(0.973)EY(0.954)KS(0.059)DAK	2,8E-227	223,44	7317600	Y708, Y713, Y715
RAS(0.051)AY(0.933)QDY(0.016)GY(0.003)Y(0.008)EY(0.982)EY(0.008)KSDAK	1,9E-202	218,72	2389500	Y705, Y713
ASAYQDY(0.01)GY(0.88)Y(0.153)EY(0.961)EY(0.963)KS(0.033)DAK	3E-202	217,89	33255000	Y708, Y711, Y713
ASAYQDY(0.992)GY(0.031)Y(0.979)EY(0.89)EY(0.105)KS(0.004)DAK	4E-202	215,83	9139900	Y705, Y710, Y711
ASAY(0.973)QDY(0.029)GY(0.988)Y(0.016)EY(0.995)EY(0.963)KS(0.035)DAK	1,2E-178	203,5	5857500	Y705, Y710, Y713, Y715
RAS(0.05)AY(0.879)QDY(0.547)GY(0.53)Y(0.487)EY(0.507)EY(0.938)KS(0.062)DAK	2,5E-175	209,01	2687600	Y705, Y715
ASAYQDY(0.845)GY(0.153)Y(0.002)EY(0.016)EY(0.958)KS(0.026)DAK	1,5E-157	203,23	85133000	Y713
ASAY(1)QDY(1)GY(0.986)Y(0.028)EY(0.979)EY(0.006)KS(0.001)DAK	2,8E-157	197,99	3648300	Y705, Y708, Y710, Y713
RAS(0.069)AY(0.917)QDY(0.016)GY(0.999)Y(0.999)EY(0.967)EY(0.033)KS(0.001)DAK	1,7E-133	189,51	1162200	Y705, Y710, Y711, Y713
AS(0.099)AY(0.9)QDY(0.003)GY(0.933)Y(0.064)EY(0.005)EY(0.987)KS(0.008)DAK	8,4E-119	175,6	1193300	Y705, Y710, Y715
ASAYQDY(0.018)GY(0.036)Y(0.237)EY(0.892)EY(0.782)KS(0.035)DAK	3,9E-103	163,61	51991000	Y711
AS(0.083)AY(0.915)QDY(0.001)GY(0.002)Y(0.04)EY(0.959)EY(0.986)KS(0.013)DAK	2,22E-88	166,09	343190	Y705, Y713, Y715
AS(0.006)AY(0.994)QDY(1)GYY(0.003)EY(0.02)EY(0.977)K	8,46E-87	169,09	3002100	Y705, Y708, Y713
AS(0.002)AY(0.998)QDY(0.046)GY(0.909)Y(0.065)EY(0.969)EY(0.011)K	9,77E-72	158,06	314100	Y705, Y710, Y713
AS(0.003)AY(0.433)QDY(0.549)GY(0.171)Y(0.883)EY(0.958)EY(0.961)KS(0.043)DAK	5,64E-49	131,94	3715600	Y711, Y713, Y715
RAS(0.1)AY(0.309)QDY(0.602)GY(0.989)Y(0.993)EY(0.742)EY(0.253)KS(0.012)DAK	2,51E-36	130,05	343500	Y710, Y711
RAS(0.23)AY(0.751)QDY(0.06)GY(0.961)Y(0.994)EY(0.861)EY(0.137)KS(0.006)DAK	6,16E-36	128,67	1198500	Y710, Y711, Y713
AS(0.005)AY(0.995)QDY(0.99)GY(0.982)Y(0.808)EY(0.239)EY(0.887)KS(0.094)DAK	3,47E-17	99,86	1130600	Y705, Y708, Y710,Y 715
RAS(0.185)AY(0.359)QDY(0.719)GY(0.873)Y(0.897)EY(0.946)EY(0.906)KS(0.115)DAK	4,82E-10	77,045	181200	Y710, Y711, Y713, Y715
AS(0.052)AY(0.947)QDY(0.798)GY(0.629)Y(0.851)EY(0.731)EY(0.852)KS(0.139)DAK	1,09E-05	49,528	169440	Y705, Y711, Y715

Supplementary Table 2. Cloning primers and plasmids

Primer name	Sequence (5' → 3')	Products
Wzabc_Fw	AACAATTCCCCTCTAGAAATAATTAATAGTG CACAGGATAATTACTCTGCC	pET21-Wza-W zb-Wzc
Wzabc_Rv	CAGTGGTGGTGGTGGTGGTGTTTCGCATC CGACTTATATTGTATTCGTA	
pET21-V_Rv	TATTTCTAGAGGGGAATTGTTATCCGCTCA	
pET21_V_Fw	CACCACCACCACCACCACTG	
Wza-strep_Fw	TGGGAGAACTTGTACTTCCAGAGCTGGAG CCACCCGCAGTTCGAAAAATGAACGGATA CAGCCAGCGACAT	pET21-Wza-strep-Wzb-Wzc
Wza-strep_Rv	AAGTACAAGTTCTCCCAATTATGAATATCAG ACGCCGTATCGGTCATGTAACGGACACCG CTAATAG	
WzcK540M_Fw	AGCCCGTCAATTGGTATGACCTTTGTCTGC GCC	Any constructs containing K540M mutation
WzcK540M_Rv	GGCGCAGACAAAGGTCATACCAATTGACG GGCT	
Wzc_ΔJR_Fw	GGTGGCGGTGGCTCGTACTCCACGCTGG GGATGATC	pET21-Wza-Wzb-$Wzc_{\Delta JR}$-strep
Wzc_ΔJR_Rv	ACGAGCCACCGCCACCAATATCGAGGTCG AGATCGTCCA	
WzcR374E_Fw	ATTGTCgaaCTGACCCGCGATGTCGAG	pET21-Wza-Wzb-Wzc_{R374E}-strep
WzcR374E_Rv	GGGTCAGttcGACAATCTCCTGCTGGGTTTT CG	
WzcE354R_Fw	GAAGACcgcAAAGCCAAACTTAACGGTCGC	pET21-Wza-Wzb-Wzc_{E354R}-strep
WzcE354R_Rv	TGGCTTTgcgGTCTTCCAGCGCCTGACG	
WzcK307E_Fw	GGAAGCAgAAGCGGTGCTCGATTCGATG	pET21-Wza-Wzb-Wzc_{K307E}-strep
WzcK307E_Rv	CACCGCTTcTGCTTCCAGCGGCAGATCA	
WzcD301R_Fw	TCTGTTcgcCTGCCGCTGGAAGCAAAAG	pET21-Wza-Wzb-Wzc_{D301R}-strep
WzcD301R_Rv	GCGGCAGgcgAACAGAATCTTTATCCTGAC GGAAGG	
Wzc_AC_mutant_Fw	gaaCTGgcggcgAAAGTTgaaCCGGCGTACCG CACGCTG	pET21-Wza-Wzb-$Wzc_{K332E,Y334A,T335A,}$

Wzc_AC_mutant_Rv	CTTTcgccgcCAGttcGGAGATTTCCGCCTCTTTAAAGGTCAG	$_{H338E}$-strep
wzabc_Cm_KO_Fw	GCTATTAATAGTGCACAGGATAATTACTCTGCCAAAGTGATAAATAAACAgccatggtccatatgaatatcctcc	*E.coli* JM109(DE3)Δ*wzabc*
wzabc_Cm_KO_Rv	CGGCGTGAACGCCTTATCCGGCCTACGGGGCGGTGCGAATGCAGGCGTCGgagcgattgtgtaggctgga	
Wzc-strep_Fw	ATCTTTATTTTCAGGGCTGGAGCCACCCGCAGTTCGAAAAATGAGATCCGGCTGCTAACAA	pET21-Wza-Wzb-Wzc-strep
Wzc-strep_Rv	GCCCTGAAAATAAAGATTCTCGCCGCTGCTTTTCGCATCCGACTTATATTCGTATTC	
Wzbc_Fw	AAAGTGATAAATAAACAATGTTTAACAACATCTTAGTTGTCTGTG	pET21-Wzb-Wzc-strep
Wzbc_Rv	TGTTTATTTATCACTTTGGCAGAGTAATTATCCT	
sfGFP-WzcJR_Fw	ggcatggatgaactctacaaaCTCGATATTGCAGTGAGCAAAAACAC	pET28-sfGFP-WzcJR
sfGFP-WzcJR_Rv	GCAGCAGTTCAGAAACTATTACGTGGAGTATTTGGTGACGGTAAACT	
Wzad87-167_Fw	CggcggcggcagcGCCTTCCGCTCACAAAAGG	pET21-Wza$_{ΔD1}$-mScarlet3-Wzb-Wzc-sfGFP
Wzad87-167_Rv	GGCgctgccgccgccGACGCGATACTGGTAATTCGC	
WzcK332E_Fw	ATCTCCgaaCTGTACACCAAAGTTCACCCG	pET21-Wza-Wzb-Wzc$_{K332E}$-strep
WzcK332E_Rv	TGTACAGttcGGAGATTTCCGCCTCTTTAAAGGT	
WzcY334A_Fw	AAGCTGgcgACCAAAGTTCACCCGGCGTA	pET21-Wza-Wzb-Wzc$_{Y334A}$-strep
WzcY334A_Rv	CTTTGGTcgcCAGCTTGGAGATTTCCGCCT	
WzcT335A_Fw	GCTGTACgcgAAAGTTCACCCGGCGTACC	pET21-Wza-Wzb-Wzc$_{T335A}$-strep
WzcT335A_Rv	AACTTTcgcGTACAGCTTGGAGATTTCCGC	
WzcH338E_Fw	CAAAGTTgaaCCGGCGTACCGCACGCTG	pET21-Wza-Wzb-Wzc$_{H338E}$-strep
WzcH338E_Rv	GCCGGttcAACTTTGGTGTACAGCTTGGAGATTTCC	
Wzc-sfGFP_Fw	TACGAATATAAGTCGGATGCGAAAGGTTCTTCTATGAGCAAAGGAGAAGAACTTTTC	

Wzc-sfGFP_Rv	CAGTGGTGGTGGTGGTGGTGTTTGTAGAG TTCATCCATGCCATG	pET21-Wza-mScarlet3-Wzb-Wzc-sfGFP
Wzc-sfGFP_V_Fw	CACCACCACCACCACCACTG	
Wzc-sfGFP_V_Rv	TTTCGCATCCGACTTATATTCGTA	
Wza-mScarlet3_Fw	TGGGAGAACTTGTACTTCCAGAGCGATAG CACCGAGGCAGTGATC	
Wza-mScarlet3_Rv	TCGAACTGCGGGTGGCTCCAGGAACCACC GGAGCCACCG	
**Plasmid name**	**description**	**source/reference**
pET21-Wza-Wzb-Wzc	Contains entire *E. coli* K12 wzabc operon	This study
pET21-Wza-strep-Wzb-Wzc	Production of Wza-strep, Wzb, and Wzc protein	This study
pET21-Wza-Wzb-Wzc-strep	Production of Wza-strep, Wzb, and Wzc-strep protein	This study
pET21-Wza-Wzb-Wzc_{K540M}-strep	Production of Wza-strep, Wzb, and Wzc_{K540M}-strep protein	This study
pET21-Wza-strep-Wzb-Wzc_{K540M}-strep	Production of Wza-strep, Wzb, and Wzc_{K540M}-strep protein	This study
pET21-Wza-Wzb-Wzc-strep	Production of Wza, Wzb, and Wzc-strep protein	This study
pET21-Wza-Wzb-Wzc_{K540M}-strep	Production of Wza, Wzb, and Wzc_{K540M}-strep protein	This study
pET21-Wzb-Wzc-strep	Production of Wzb and Wzc-strep protein	This study
pET21-Wzb-Wzc_{K540M}-strep	Production of Wzb and Wzc_{K540M}-strep protein	This study
pKD3	The cat cassette	[3]
pKD46	the λ red recombineering	[3]
pET28-Avitag-Wzc_{JR}	Production of Avitagged Jellyroll domain of Wzc	This study
pET28-Avitag-Wzc_{Peri}	Production of Avitagged periplasmic domain of Wzc	This study
pACYC-BirA	Used in vivo biotinylation of Wzc_{JR} and Wzc_{Peri}	Gift from Dr. Joop Van den Heuvel
pET21-Wza-Wzb-Wzc_{R374E}-strep	Production of Wza, Wzb, and Wzc_{R374E}-strep protein	This study

pET21-Wza-Wzb-Wzc$_{E354R}$-strep	Production of Wza, Wzb, and Wzc$_{E354R}$-strep protein	This study
pET21-Wza-Wzb-Wzc$_{D301R}$-strep	Production of Wza, Wzb, and Wzc$_{D301R}$-strep protein	This study
pET21-Wza-Wzb-Wzc$_{K307E}$-strep	Production of Wza, Wzb, and Wzc$_{K307E}$-strep protein	This study
pET21-Wza-Wzb-Wzc$_{K332E,Y334A,T335A,H338E}$-strep	Production of Wza, Wzb, and Wzc$_{K332E,Y334A,T335A,H338E}$-strep protein	This study
pET21-Wza-Wzb-Wzc$_{\Delta JR}$-strep	Production of Wza, Wzb, and Wzc$_{\Delta JR}$-strep protein	This study
pET21-Wza-mScarlet3-Wzb-Wzc-sfGFP	Production of Wza-mScarlet3, Wzb, and Wzc-sfGFP	This study
pET21-Wza$_{\Delta D1}$-mScarlet3-Wzb-Wzc-sfGFP	Production of Wza$_{\Delta D1}$-mScarlet3, Wzb, and Wzc-sfGFP	This study
pET28-sfGFP-Wzc$_{JR}$	Production of sfGFP-Wzc$_{JR}$ fusion	This study
pET21-Wza-Wzb-Wzc$_{K332E}$-strep	Production of Wza, Wzb, and Wzc$_{K332E}$-strep protein	This study
pET21-Wza-Wzb-Wzc$_{Y334A}$-strep	Production of Wza, Wzb, and Wzc$_{Y334A}$-strep protein	This study
pET21-Wza-Wzb-Wzc$_{T335A}$-strep	Production of Wza, Wzb, and Wzc$_{T335A}$-strep protein	This study
pET21-Wza-Wzb-Wzc$_{H338E}$-strep	Production of Wza, Wzb, and Wzc$_{H338E}$-strep protein	This study
**Strain name**	**Genotype or description**	**source/reference**
E.coli Top10	F- mcrA Δ(mrr-hsdRMS-mcrBC) φ80lacZΔM15 ΔlacX74 nupG recA1 araD139 Δ(ara-leu)7697 galE15 galK16 rpsL(Str^R) endA1 λ	Invitrogen
E.coli JM109 (DE3)	*end*A1, *rec*A1, *gyr*A96, *thi, hsd*R17 (r_k^-, m_k^+), *rel*A1, *sup*E44, λ–, Δ(*lac-pro*AB), [F´, *tra*D36, *pro*AB, *lac*I^{q}ZΔM15], lDE3	Promega
E.coli JM109 (DE3) Δ*wzabc*	*E.coli* JM109 (DE3) Δwzabc::cat	This study
E.coli BL21 Star (DE3)	F$^-$*omp*T *hsd*SB (r_B–, m_B–) *gal dcm rne*131 (DE3)	Thermo Fisher Scientific

Supplementary Table 3.1 Cryo-EM data collection, refinement and validation statistics

	Wza_C1 (EMD-53598; PDB 9R60)	Wza_C8 (EMD-53599; PDB 9R61)	Wzc_{K540M}_C1 (EMD-53600; PDB 9R62)	Wzc_{K540M}_C4 (EMD-53601; PDB 9R63)	Wza-Wzc_{K540M}_C1 (Conf 0) (EMD-53602;PDB 9R64)	Wza-Wzc_{K540M}_C8 (Conf 0) (EMD-53603; PDB 9R65)
**Data collection and processing**						
Magnification	130k	130k	130k	130k	130k	130k
Voltage (kV)	200	200	200	200	200	200
Electron exposure ($e^{-}/Å^2$)	40	40	40	40	40	40
Defocus range (μm)	–0.6 to –2.0	–0.6 to –2.0	–0.6 to –2.0	–0.6 to –2.0	–0.6 to –2.0	–0.6 to –2.0
Pixel size (Å)	0.91	0.91	0.91	0.91	0.91	0.91
Symmetry imposed	C1	C8	C1	C4	C1	C8
Initial particle images (no.)	1,581,132	1,581,132	256,861	256,861	363,741	363,741
Final particle images (no.)	125,938	125,938	128,284	128,284	207,837	207,837
Map resolution (Å)	3.0	2.7	3.4	3.2	3.5	3.2
FSC threshold	0.143	0.143	0.143	0.143	0.143	0.143
**Refinement**						
Initial model used	Alphafold3	Alphafold3	Alphafold3	Alphafold3	Alphafold3	Alphafold3
Model resolution (Å)	3.1	2.8	3.6	3.3	3.6	3.3

FSC threshold	0.5	0.5	0.5	0.5	0.5	0.5
Map sharpening *B* factor (Å^2)	–85.3	–109.9	–97.8	–125.1	–82.5	–132.5
Model composition						
Non-hydrogen atoms	21,616	21,616	40,312	40,312	63,984	63,984
Protein residues	2,784	2,784	5,168	5,168	8,208	8,208
Ligands	0	0	8 ADP-Mg^{2+}	8 ADP-Mg^{2+}	8 ADP-Mg^{2+}	8 ADP-Mg^{2+}
B factors (Å^2)						
Protein	54.43	30.49	61.37	91.68	56.29	92.81
Ligand	-	-	33.37	45.98	118.79	141.99
R.m.s. deviations						
Bond lengths (Å)	0.005	0.005	0.004	0.005	0.004	0.004
Bond angles (°)	1.015	1.034	0.979	1.006	0.945	0.973
Validation						
MolProbity score	1.15	1.03	1.35	1.39	1.22	1.11
Clash score	2.41	1.81	2.94	2.71	3.48	2.21
Poor rotamers (%)	1.05	0.88			0.04	0.07
Ramachandran plot						
Favored (%)	97.43	97.54	96.11	95.23	97.64	97.45
Allowed (%)	2.57	2.46	3.89	4.77	2.21	2.42
Disallowed (%)	0.00	0.00	0.00	0.00	0.15	0.14

Supplementary Table 3.2 Cryo-EM data collection, refinement and validation statistics

	**Wza-Wzc$_{K540M}$ _C1 (Conf Ⅰ) (EMD-53604; PDB 9R66)**	**Wza-Wzc$_{K540M}$ _C8 (Conf Ⅰ) (EMD-53605; PDB 9R67)**	**Wza-Wzc$_{K540M}$ _C1 (Conf Ⅱ) (EMD-53606; PDB 9R68)**	**Wza-Wzc$_{K540M}$ _C8 (Conf Ⅱ) (EMD-53607; PDB 9R69)**	**Wza-Wzc$_{K540M}$ _C1 (Conf Ⅲ) (EMD-53608; PDB 9R6A)**	**Wza-Wzc$_{K540M}$_ C1 (Conf Ⅳ) (EMD-53609; PDB 9R6B)**	**Wza-Wzc$_{K540M}$ _C1 (Conf Ⅴ) (EMD-53610; PDB 9R6C)**
**Data collection and processing**							
Magnification	130k	130k	130k	130k	130k	130k	130k
Voltage (kV)	200	200	200	200	200	200	200
Electron exposure (e^{-}/Å^2)	40	40	40	40	40	40	40
Defocus range (μm)	–0.6 to –2.0	–0.6 to –2.0	–0.6 to –2.0	–0.6 to –2.0	–0.6 to –2.0	–0.6 to –2.0	–0.6 to –2.0
Pixel size (Å)	0.91	0.91	0.91	0.91	0.91	0.91	0.91
Symmetry imposed	C1	C8	C1	C8	C1	C1	C1
Initial particle images (no.)	468,471	468,471	468,471	468,471	468,471	468,471	468,471
Final particle images (no.)	199,355	199,355	26,950	26,950	27,501	40,425	59,128
Map resolution (Å)	3.8	3.4	5.8	4.2	6.3	4.6	6.7
FSC threshold	0.143	0.143	0.143	0.143	0.143	0.143	0.143
**Refinement**							
Initial model used	Alphafold3	Alphafold3	Alphafold3	Alphafold3	Alphafold3	Alphafold3	Alphafold3

Model resolution (Å)	4.0	3.6	7.2	4.5	7.6	8.0	9.4
FSC threshold	0.5	0.5	0.5	0.5	0.5	0.5	0.5
Map sharpening *B* factor (Å^2)	–100.4	–132.5	–203.9	–103.0	–260.5	–76.4	–377.8
Model composition							
Non-hydrogen atoms	63,976	63,976	64,072	64,072	64,256	62,068	86,248
Protein residues	8,208	8,208	8,232	8,232	8,248	7,968	11,072
Ligands	8 ADP	8 ADP	8 ADP	8 ADP	8 ADP	8 ADP	8 ADP
B factors (Å^2)							
Protein	88.87	79.80	274.43	202.25	147.39	80.52	74.13
Ligand	132.20	130.52	172.32	89.91	89.91	130.52	69.83
R.m.s. deviations							
Bond lengths (Å)	0.006	0.004	0.004	0.003	0.015	0.013	0.023
Bond angles (°)	1.029	0.987	0.815	0.549	1.854	1.833	2.138
Validation							
MolProbity score	1.43	1.26	2.01	1.56	1.51	1.10	1.51
Clash score	5.31	3.59	16.33	5.26	0.91	0.18	1.74
Poor rotamers (%)	0.06	0.10	0.00	0.00	2.92	1.81	2.33
Ramachandran plot							
Favored (%)	97.19	97.42	95.73	95.90	94.34	95.23	95.61
Allowed (%)	2.68	2.48	3.69	3.81	5.32	4.38	3.84
Disallowed (%)	0.14	0.10	0.59	0.29	0.34	0.39	0.55

Supplementary Table 4. Glycans from the RayBio® Glycan Array 100 Recognized by the JR Domain

Glycan ID	Structure / Motif	Category	Functional Interpretation
2	β-Gal-Sp	Monosaccharide	Terminal galactose
3	α-Man-Sp	Monosaccharide	Mannose residue typical of N-glycan cores
5	α-Rha-Sp	Monosaccharide	Rhamnose
8	Tobramycin	Aminoglycoside	Positively charged antibiotic; likely electrostatic binding, not specific carbohydrate recognition.
9	Galβ1–3GlcNAcβ-Sp (LacNAc)	Disaccharide	N-acetyllactosamine; key N-/O-glycan core motif
28	4-P-GlcNAcβ1–4Manβ-Sp	Phosphorylated Disaccharide	Mannose-phosphate–like motif
39	GlcNAcβ1–6(Galβ1–3)GalNAcα-O-Ser-Sp4	Core 2 O-Glycan	Mucin-type O-glycan core extension
52	β-D-GlcA-Sp	Acidic Monosaccharide	Glucuronic acid; negatively charged sugar
53	Galβ1–4(6S)GlcNAcβ-Sp	Sulfated Disaccharide	6-O-sulfated LacNAc; selectin ligand–like motif
56	Sisomicin sulfate	Aminoglycoside	Highly cationic; reproducible electrostatic interaction rather than true glycan binding.
85	D-pentamannuronic acid-β-Sp1	Acidic Polysaccharide	Uronic acid polymer; mimics
97	Kanamycin sulfate	Aminoglycoside	Poly-cationic; charge-driven interaction
99	Neomycin trisulfate	Aminoglycoside	Strongly charged; electrostatic or ionic interaction.

Note: Only samples with all three tested concentrations showing fluorescence intensity >500 were considered.

SUPPLEMENTARY REFERENCES

1. Yang, Y. *et al.* The molecular basis of regulation of bacterial capsule assembly by Wzc. *Nat. Commun.* **12**, 4349 (2021).
2. Wiseman, B., Nitharwal, R. G., Widmalm, G. & Högbom, M. Structure of a full-length bacterial polysaccharide co-polymerase. *Nat. Commun.* **12**, 369 (2021).
3. Datsenko, K. A. & Wanner, B. L. One-step inactivation of chromosomal genes in Escherichia coli K-12 using PCR products. *Proc. Natl. Acad. Sci. USA* **97**, 6640–6645 (2000).
